# A Case of Cystic Neutrophilic Granulomatous Mastitis in Which *Mycobacteroides abscessus* Was Detected

**DOI:** 10.70352/scrj.cr.24-0115

**Published:** 2025-02-07

**Authors:** Hirokazu Yamazaki, Yasushi Ito, Keigo Goto, Masako Kasami

**Affiliations:** 1 Department of Breast Surgery, Koga Community Hospital, Yaizu, Shizuoka, Japan; 2 Department of Breast Surgery, Iwata City Hospital, Iwata, Shizuoka, Japan; 3 Department of Pathology, Iwata City Hospital, Iwata, Shizuoka, Japan

**Keywords:** cystic neutrophilic granulomatous mastitis, *Mycobacteroides abscessus*, antibacterial therapy, drainage, ultrasound-guided vacuum-assisted biopsy, magnetic resonance imaging

## Abstract

**INTRODUCTION:**

Cystic neutrophilic granulomatous mastitis (CNGM) is characterized by granulomas with cysts that sometimes contain bacteria in the lumen, a surrounding neutrophilic infiltrate, and Langhans giant cells. There are no universally accepted diagnostic criteria for CNGM. *Corynebacterium kroppenstedtii*, a Gram-positive bacillus, has been reported to cause several infections, but the exact cause remains unclear. We report our experience with a case of CNGM, thought to be due to a rare *Mycobacteroides abscessus* infection.

**CASE PRESENTATION:**

We report the case of a 36-year-old Japanese woman with granulomatous mastitis due to *Mycobacteroides abscessus* who had not undergone surgery and was not immunosuppressed. She presented with a chief complaint of pain and swelling in her left breast for 1 month. Mammography showed asymmetrical focal increased density, and ultrasonography showed an irregular hypoechoic area in the left 3 o’clock position. Contrast-enhanced magnetic resonance imaging showed segmental non-mass-enhancement. Ultrasound-guided vacuum-assisted biopsy with pathology revealed granulomatous mastitis. Ziehl–Neelsen staining revealed red-staining bacilli. The patient was followed up for observation because her breast pain had decreased after the examination, and there was no redness or fever. However, the breast pain has not completely disappeared, and intermittent purulent discharge from the biopsy site persisted for 5 months. Hence, two 12-Fr drains were inserted along the ductal dilatation-like hypoechoic area. Imipenem, amikacin, and clarithromycin were administered for 8 days. After 8 days of this therapy, the patient developed a drug-associated rash; therefore, antimicrobial therapy was discontinued, and the drains were removed. Her symptoms improved, and magnetic resonance imaging after 1 month showed that the previous imaging findings in her left breast had disappeared. At the time of writing, 18 months have passed since treatment, and no recurrence has been observed.

**CONCLUSIONS:**

We experienced a rare case of CNGM associated with *Mycobacteroides abscessus*. This case suggests that a combination of drainage and antimicrobial therapy may shorten the duration of antimicrobial therapy in CNGM.

## Abbreviations

CNGM
: cystic neutrophilic granulomatous mastitis
COVID-19
: coronavirus disease 2019
MRI
: magnetic resonance imaging
NTM
: non-tuberculous mycobacteria

## INTRODUCTION

Cystic neutrophilic granulomatous mastitis (CNGM) is a type of granulomatous mastitis characterized by granulomas with cysts. Bacteria may be present in the lumen, with a surrounding neutrophilic infiltrate, and Langhans giant cells, which are found in less than 1% of breast specimens.^1,2)^ CNGM is usually seen in women of childbearing age who have breastfed. Granulomatous mastitis is usually associated with the Gram-positive rod, *Corynebacterium kloppenstedii*, and rarely with the non-tuberculous mycobacteria (NTM), *Mycobacteroides abscessus*.^1)^ CNGM caused by *Mycobacteroides* infection is a reported cause of breast tissue infection after cosmetic surgery and in immunosuppressed individuals.^3,4)^

## CASE PRESENTATION

**Fig. 1:**
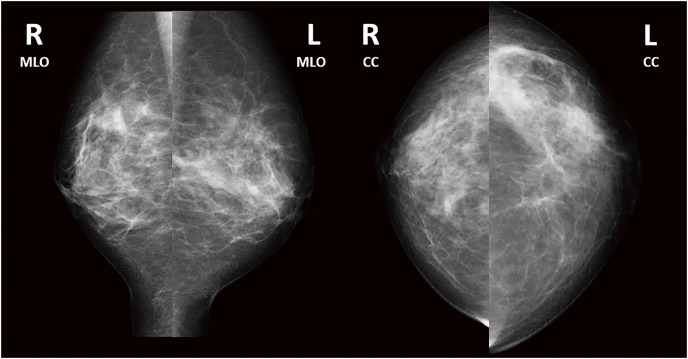
Mammography findings in a case of cystic neutrophilic granulomatous mastitis caused by *Mycobacteroides abscessus*. The image shows an asymmetrical focal increased density in the left breast. No evidence of tumor calcification is visible. CC, craniocaudal; L, left; MLO, mediolateral oblique view; R, right

**Fig. 2:**
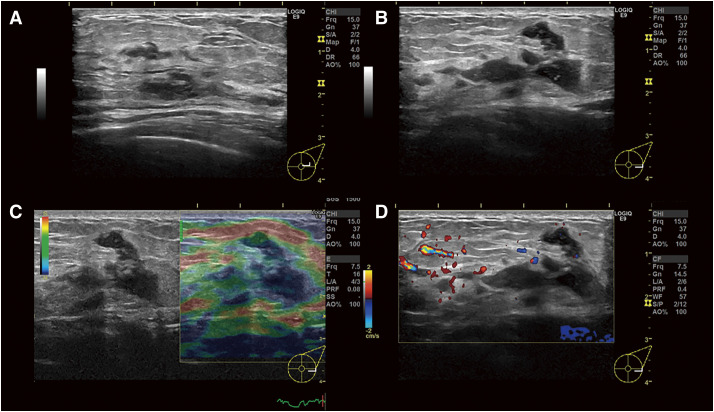
Ultrasonographic imaging findings in a case of cystic neutrophilic granulomatous mastitis caused by *Mycobacteroides abscessus*. (**A** and **B**) Ultrasonographic images showing an irregular hypoechoic area extending over the map to replace the mammary tissue in the left breast at the left 3 o’clock position. The hypoechoic area extends partially to just below the skin. (**C**) The Tsukuba elasticity score was 5, suggesting malignancy. (**D**) Color Doppler image showing increased blood flow signals around dilated ducts.

**Fig. 3:**
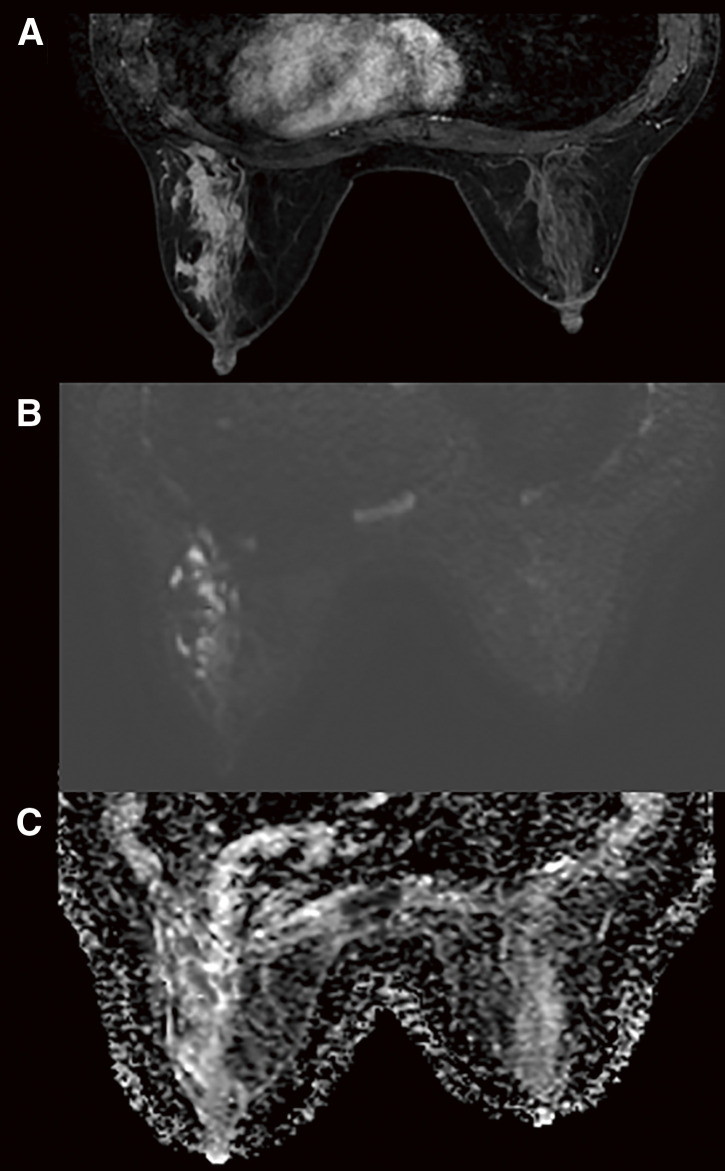
Contrast-enhanced MRI in a case of cystic neutrophilic granulomatous mastitis caused by *Mycobacteroides abscessus*. (**A**–**C**) Contrast-enhanced MRI showing a segmental non-enhanced area measuring approximately 36 × 29 × 89 mm. The contrast pattern increased progressively. Diffusion-weighted imaging shows a high signal intensity, and apparent diffusion coefficient mapping shows some low-intensity signals, strongly suspicious for DCIS or invasive carcinoma with a predominant intraductal component. DCIS, ductal carcinoma in situ; MRI, magnetic resonance imaging.

**Fig. 4:**
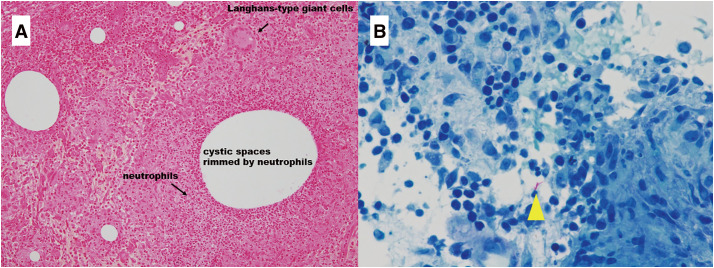
Histopathological findings in a case of cystic neutrophilic granulomatous mastitis, associated with infection caused by *Mycobacteroides abscessus*. (**A**) Breast core biopsy results showing large (right side) and small (surrounding the large cyst)suppurative granulomas that are composed of central lipid vacuoles (cysts) rimmed by neutrophils and an outer cuff of epithelioid histiocytes including Langhans-type giant cells (hematoxylin and eosin, ×10). (**B**) Ziehl–Neelsen staining showing red-stained bacilli (yellow arrowhead) (×100). Granulomatous tissue is seen on the right side.

**Fig. 5:**
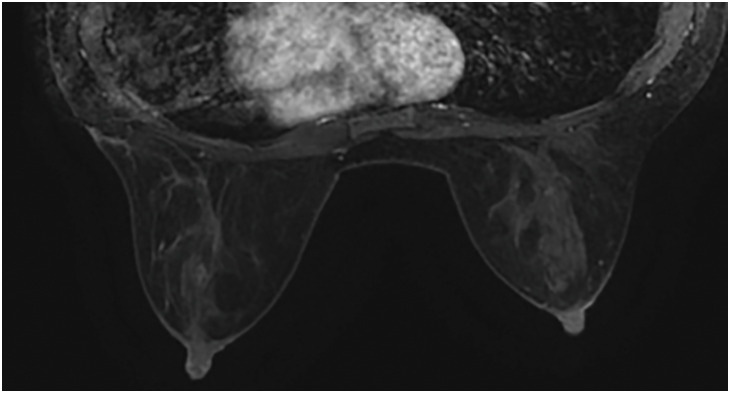
MRI 1-month post-antimicrobial therapy showing that the previous findings in the left breast had disappeared.

A 36-year-old Japanese woman presented with a chief complaint of pain and swelling in her left breast. She was healthy, with no history of surgery and no immunodeficiency. She did not have a history of fever, and there were no respiratory symptoms. She had experienced 2 pregnancies and 2 deliveries and had been breastfeeding until 18 months before presentation. On palpation, an induration with indistinct borders was palpated at 3 o’clock in the left breast. Mammography revealed asymmetrical focal increased density in the left breast (**Fig. 1**). Ultrasonography revealed an irregular hypoechoic area in the left 3 o’clock direction, and the hypoechoic area extended partially to just below the skin. The part of irregular hypoechoic area was considered a segmental ductal dilatation. The internal echo was solid component without fluidity. In addition, Color Doppler ultrasonography revealed increased blood flow signals around dilated ducts (**Fig. 2**). Contrast-enhanced magnetic resonance imaging (MRI) revealed segmental non-mass-enhancement in an area measuring approximately 36 × 29 × 89 mm (**Fig. 3A–3C**). The contrast pattern increased progressively. Diffusion-weighted imaging revealed high-intensity signals, and apparent diffusion coefficient mapping revealed some low-intensity signals, strongly suggestive of ductal carcinoma in situ or invasive carcinoma with a predominant intraductal component. Ultrasound-guided vacuum-assisted biopsy and histopathology revealed several central lipid vacuoles, rimmed by neutrophilic infiltrate, and an outer cuff of epithelioid histiocytes with Langhans giant cells (**Fig. 4A**). No palisade arrangement of histiocytes was seen. Gram positive bacilli bacteria were found in these cysts (not shown). These findings are characteristic of CNGM. General bacterial and fungal cultures of the collected tissue were negative. The patient was followed up for observation because her breast pain had decreased after the examination, and there was no redness or fever. However, intermittent purulent discharge persisted from the biopsy site. After 1 month of follow-up, culture of the biopsy tissue confirmed the development of *Mycobacteroides abscessus*, and Ziehl–Neelsen staining revealed red-staining bacilli (**Fig. 4B**). On the basis of a previous case report, two 12-Fr drains were inserted along the ductal dilatation site in the left breast under ultrasound guidance. There was minimal drainage of pus from the drains. Due to the presence of refractory symptoms for 5 months, clarithromycin, amikacin, and imipenem were administered simultaneously considering the literature. The results of the minimal inhibitory concentration of the culture test showed promising sensitivity to all of them. Drainage of the drain was initially brownish-purulent viscous but gradually became serous after antimicrobial administration was started. After 8 days of intravenous antibacterial therapy (imipenem, amikacin, clarithromycin), the patient developed a fever of 39.2°C, generalized erythema, and an oral mucosal rash. Drug eruption was suspected, and treatment was discontinued. A skin biopsy was performed, and the findings were consistent with drug eruption. The patient’s symptoms improved after drug discontinuation, and we chose not to restart either intravenous or oral antimicrobials. MRI 1 month post-antimicrobial therapy showed that the previous findings in the left breast had disappeared (**Fig. 5**). After discontinuing the antimicrobials, the patient remained under observation. At the time of writing, 18 months have passed since treatment, and no recurrence has been observed.

## DISCUSSION

There are no specific clinical or imaging findings specific to granulomatous mastitis caused by Mycobacterial infection. Furthermore, there are no universally accepted diagnostic criteria for CNGM. Treatment options for granulomatous mastitis may include observation, antibacterial agents, steroids, incisional drainage, and excision. A recent article proposed subdividing treatment into cases related to autoimmunity, cases related to infection, and idiopathic cases.^2)^ CNGM is often associated with *Corynebacterium*, but rarely with NTM, which requires careful Gram staining and microbiological examination.^1,2)^ Drainage and antimicrobial therapy are recommended when abscesses form, and the indications for steroid administration should be judged carefully.^2)^ It is appropriate to first confirm whether there are any histological images suspicious for *Mycobacterium tuberculosis* or *Mycobacteroides abscessus*.

*Mycobacteroides abscessus* can sometimes be proven by Ziehl–Neelsen staining of biopsy specimens,^4)^ and it is useful to ask the pathologist to perform Ziehl–Neelsen staining when bacteria are suspected. On the other hand, many cases are not detected the bacilli by the staining. It is also appropriate to confirm *Mycobacteroides tuberculosis* or *Mycobacteroides abscessus* in culture. *Mycobacteroides abscessus* is the most pathogenic and rapidly multiplying Gram-positive mycobacterium and is classified as an NTM.^5)^ *Mycobacteroides abscessus* is frequently responsible for contaminated traumatic skin wounds and postoperative soft tissue infections.^4,6,7)^ Granulomatous mastitis is most common in women with a history of lactation and may be transmitted via small nipple trauma from suckling. Villanueva et al. reported a high incidence of post-injection site infections due to *Mycobacteroides abscessus*, with incubation periods ranging from 7 to 121 days.^8)^ We identified 5 reports of granulomatous mastitis caused by *Mycobacteroides abscessus* in women with no history of trauma or surgery.^9–13)^ To our knowledge, ours is the first report of CNGM in a Japanese patient. A literature review indicated that CNGM affects women ranging in age from 27 to 54 years (**Table 1**). CNGM is typically reported in women of childbearing age, and the condition occurred in our patient 18 months after lactation. Clinical manifestations are a mass, breast pain, swelling, and redness.^14)^ Radiological findings of CNGM are reported rarely,^14)^ with the most common ultrasonographic findings of a mass (72.2%), followed by dilated ducts (11.1%), abscess (5.6%), edema (5.6%), and fluid retention (5.6%). Contrast-enhanced MRI was very useful in our case to diagnose the site of granulomatous mastitis and compare findings before and after treatment. The differential diagnosis of breast cancer was raised in the imaging studies for our patient, and histology is considered important to obtain a diagnosis.

**Table 1:** Imaging findings and treatment of CNGM reports

	Year of publication	Age (years)	Country	History	Mammography	Ultrasonography	MRI	Mass size (cm)	Surgery	Antimicrobials (Duration)
Patel et al.^9)^	2021	34	India	3 months	N/A	N/A	N/A	10 × 7	Incision and drainage	Clarithromycine, amikacin(2 weeks) → Clarithromycine(8 weeks)
Wang et al.^10)^	2016	29	China	40 days	N/A	Mass	N/A	1.7 × 1	N/A	Doxycycline, clindamycin, amoxicillin-clavulanic acid (10 days) → Rifampicin, isoniazid, pyrazinamide(6 months)
Yasar et al.^11)^	2011	39	Türkiye	3 years	Local asymmetric density	Cystic lesion	Cystic abscess	4 × 2.5	N/A	Linezolid (2 months), clarithromycin (4 months), amikacin (3 weeks)
Urgancı et al.^12)^	2010	27	Türkiye	3 weeks	N/A	Solid lesion	Cystic, or necrotic lesion	8 × 8	N/A	Clarithromycine(6 weeks)
Pasticci et al.^13)^	2009	54	Italy	72 hours	N/A	N/A	N/A	9 × 6	N/A	Cefotaxime (2 weeks) →Cefotaxime (3 weeks), ciprofloxacin (3 weeks) →Imipenem, clarithromycin(4.5 months)
Present study	2024	36	Japan	1 month	Local asymmetric density	Ductal dilatation	Segmental non mass enhancement	9 × 4	Incision and drainage	Imipenem, amikacin, clarithromycin (8 days)

CNGM, cystic neutrophilic granulomatous mastitis.

Detection of *Mycobacteroides abscessus* in cultures of common abscesses can be difficult because of the organisms’ long growth period. Isolation of the causative organism on Löwenstein–Jensen medium is most rapid.^11)^ Not infrequently, clinicians initiate empirical antimicrobial therapy for patients. As a result of failing to identify the pathogen, the clinician has no choice but to initiate incorrect treatment based on their experience.

Clarithromycin was successful as a treatment for CNGM in many reports and should be included in combination therapy^11)^ (**Table 1**). Resistance rates for clarithromycin are up to 20%, while those for cefoxitin and amikacin are both 10%.^15)^ Antimicrobial agents used in previous studies comprise rifampicin, isoniazid, pyrazinamide, imipenem, amikacin, cefoxitin, and fluoroquinolones, and all patients recovered after long-term treatment (**Table 1**).

Invasive procedure is an important tool in treating Granulomatous mastitis.^16)^ A combined treatment approach that comprises skilled surgical drainage, debridement, and long-term (≥ 3 months) treatment with a combination of antimicrobials is considered the optimal treatment method.^11)^ In the present case, the patient was in remission after drainage and antimicrobial therapy. Although the duration of antimicrobial therapy was short (8 days) owing to symptoms that were considered associated with a drug eruption, MRI 1 month after treatment confirmed remission, and no recurrence has been observed 18 months after treatment.

In 1981, Carmalt and Ramsey-Stewart proposed the following five features of granulomatous mastitis,^17)^ but there are no clear diagnostic criteria. We propose the following diagnostic criteria from this case report. We propose the following diagnostic criteria for CNGM: (1) childbearing age, (2) refractory mastitis, (3) histopathologic findings of granulomas with Langhans-type giant cells and cystic areas, and (4) hypoechoic areas requiring ultrasound imaging to exclude breast cancer. Further studies in more patients are needed.

## CONCLUSIONS

We experienced a case of granulomatous mastitis associated with *Mycobacteroides abscessus*, an NTM, which has been reported rarely. This case suggests that a combination of drainage and antimicrobial therapy may shorten the duration of antimicrobial therapy.

## ACKNOWLEDGMENTS

We thank Jane Charbonneau, DVM, from Edanz (https://jp.edanz.com/ac) for editing a draft of this manuscript.

## DECLARATIONS

### Funding

This study did not receive any financial support.

### Availability of data and materials

Not applicable.

### Ethics approval and consent to participate

Ethics approval is not required for case reports, in our institution. Written consent to participate is obtained from the patient.

### Consent for publication

Written informed consent was obtained from the patient for the publication of this case report and the accompanying images. A copy of the written consent is available for review by the Editor-in-Chief of the journal.

### Competing interests

The authors declare that they have no conflicts of interest.
